# Supplementary material for: Variation in in-hospital mortality and its association with percutaneous coronary intervention-related bleeding complications: A report from nationwide registry in Japan
Source: PLoS One. 2021 Dec 13;16(12):e0261371. doi: 10.1371/journal.pone.0261371 (PMC8668123; doi:10.1371/journal.pone.0261371)
Supplement: Attachment — Submitted filename: Response to Reviewers.docx [file pone.0261371.s001.docx]

Response to the Reviewer #1

*Reviewer #1*

*Sawayama and colleagues have demonstrated a prognostic value of PCI-related bleeding complications. The study is of interest and timely, albeit with the inherent limitations of an observational retrospective study, as the need for more studies on the role of PCI-related bleeding is needed. I have a few questions/clarifications that will be helpful in making it a stronger study:*

*1) The definition of bleeding complications in the current study was different from standardized definitions as the authors highlighted, but it is not clear how many patients had a fatal bleeding.*

Response

We appreciate the comment from the reviewer. The J-PCI registry’s definition of bleeding complications is largely equivalent to Bleeding Academic Research Consortium (BARC) 3A-C or above. Previous studies demonstrated that the ratio of fatal bleeding among patients with BARC 3A-C or above was approximately 10% (Vranckx P, et al. J Am Coll Cardiol 2016, Ratcovich H, et al. Am J Cardiol 2021, Gargiulo G, et al. J Am Coll Cardiol 2018). Unfortunately, direct information on fatal bleeding was not available on the registry, but we believe the rate of fatal bleeding would be roughly the same for our study. We acknowledge that the causality between bleeding complications and in-hospital death cannot be proven; thus, we have added the descriptions in the Discussion section as follows:

**Discussion**

“… Second, we cannot determine the causality between bleeding complications and death during hospitalization. However, failure to rescue, defined by all-cause death after major perioperative complications, is well established as an indicator of the surgical quality of care [19]. The results in our study were equivalent to this, and this concept may be applied to PCI. Third, blood transfusion practices and thresholds vary between hospitals [25], which may have affected our results. Fourth, the number of variables in the J-PCI registry is limited and measured or unmeasured confounders were present.”

(Page 16, line 260–266, revised manuscript)

*2) Haemoglobin and platelet values are not reported as the definition of Chronic Kidney Disease (eFGR < 60 ?).*

Response

We had no data for platelet values in the J-PCI registry, but have added hemoglobin levels in Table 1 in the revised manuscript. CKD was defined as the presence of proteinuria, and/or a serum creatinine level ≥1.3 mg/dL, and/or an estimated glomerular filtration rate level ≤60 mL/min/1.73 m^2^. Based on these points, we have modified the descriptions in the Results section as follows:

**Results**

**Table 1. Patient characteristics**

|  | **Overall** | **Very low mortality** | **Low mortality** | **Medium mortality** | **High mortality** | **Very high mortality** | ***P* value** |
| --- | --- | --- | --- | --- | --- | --- | --- |
|  |  |  |  |  |  |  |  |
| Cardiogenic shock within 24 hours | 13,419 (3.5) | 3,568 (3.3) | 2,710 (3.7) | 2,481 (3.6) | 2,513 (3.5) | 2,147 (3.1) | <0.001 |
| Baseline hemoglobin, g/dL | 13.2 ± 2.0 | 13.1 ± 2.0 | 13.2 ± 2.1 | 13.2 ± 2.0 | 13.2 ± 2.1 | 13.2 ± 2.1 | 0.002 |
|  |  |  |  |  |  |  |  |
| DCA | 2,256 (0.58) | 746 (0.70) | 619 (0.84) | 446 (0.65) | 198 (0.28) | 247 (0.36) | <0.001 |

Values are expressed as means ± standard deviation or numbers (%). Chronic kidney disease was defined as the presence of proteinuria, and/or a serum creatinine level ≥1.3 mg/dL, and/or an estimated glomerular filtration rate level ≤60 mL/min per 1.73 m^2^.

(Page 10, line 149–151, revised manuscript)

*3) It is not reported what type of intensive care unit (ICU) was present (cardiac surgery, neurosurgery) in the different hospitals: different ICUs means different types of treatment.*

Response

We agree it is also important in dealing with clinical outcomes such as in-hospital death. In Japan, the ICU is a place to treat patients with critical acute dysfunction, regardless of internal medicine or surgery. According to the report from Ministry of Health, Labour and Welfare in 2017 (https://www.e-stat.go.jp/dbview?sid=0003289748), the number of hospitals advocating ICU, coronary or cardiac care unit (CCU), and stroke care unit (SCU) is 713, 287, and 162, respectively. Other high care units, such as neurosurgical care unit, are practically very few and unreported. Some hospitals have multiple care units (e.g., ICU and SCU, etc), whereas others only have an ICU. Since the name varies from hospital to hospital, the J-PCI registry collects data under the name ICU. There is no data, but it is assumed that most of them mean CCU.

*4) I think it is important to know what kind of antiplatelet agents and what kind of anticoagulant agents were used; moreover, how many patients were in triple (DAPT + anticoagulant) antithrombotic therapy?*

Response

We agree it is important in addressing the clinical outcome of bleeding complications, and we have added the information about status of antithrombotic therapy in Table 1 in the revised manuscript.

**Results**

**Table 1. Patient characteristics**

|  | **Overall** | **Very low mortality** | **Low mortality** | **Medium mortality** | **High mortality** | **Very high mortality** | ***P* value** |
| --- | --- | --- | --- | --- | --- | --- | --- |
|  | | |  |  |  |  |  |
| **Antithrombotic agents given at time of PCI** | | |  |  |  |  |  |
| Antiplatelet agents | 357,447 (92) | 97,149 (91) | 65,072 (89) | 63,565 (92) | 67,774 (95) | 63,887 (93) | <0.001 |
| Aspirin | 345,703 (89) | 93,953 (88) | 62,893 (86) | 61,652 (89) | 65,597 (92) | 61,608 (90) | <0.001 |
| Clopidogrel | 135,698 (35) | 37,721 (35) | 24,565 (34) | 26,661 (39) | 24,292 (34) | 22,459 (33) | <0.001 |
| Prasugrel | 191,263 (49) | 50,054 (47) | 35,993 (49) | 31,079 (45) | 37,380 (52) | 36,757 (54) | <0.001 |
| Ticagrelor | 468 (0.1) | 219 (0.2) | 94 (0.1) | 68 (0.1) | 53 (0.1) | 34 (0.05) | <0.001 |
| Anticoagulant agents | 26,309 (6.8) | 6,694 (6.3) | 4,950 (6.8) | 4,962 (7.2) | 5,129 (7.2) | 4,574 (6.7) | <0.001 |
| Warfarin | 10,378 (2.7) | 2,793 (2.6) | 1,972 (2.7) | 1,805 (2.6) | 2,014 (2.8) | 1,794 (2.6) | <0.001 |
| Dabigatran | 1,410 (0.4) | 348 (0.3) | 260 (0.4) | 297 (0.4) | 236 (0.3) | 269 (0.4) | 0.01 |
| Rivaroxaban | 5,004 (1.3) | 1,326 (1.2) | 834 (1.1) | 907 (1.3) | 1,026 (1.4) | 911 (1.3) | <0.001 |
| Apixaban | 5,388 (1.4) | 1,260 (1.2) | 1,078 (1.5) | 1,110 (1.6) | 1,037 (1.5) | 903 (1.3) | <0.001 |
| Edoxaban | 4,128 (1.1) | 1,046 (1.0) | 824 (1.1) | 799 (1.2) | 795 (1.1) | 664 (1.0) | 0.051 |
| Dual antiplatelet therapy | 316,061 (81) | 84,998 (80) | 58,456 (80) | 55,841 (81) | 59,763 (84) | 57,003 (83) | <0.001 |
| Triple therapy* | 19,205 (4.9) | 4,623 (4.3) | 3,727 (5.1) | 3,550 (5.1) | 3,951 (5.5) | 3,354 (4.9) | <0.001 |
|  |  |  |  |  |  |  |  |

*Triple therapy indicates an anticoagulant agent plus dual antiplatelet therapy.

(Page 10, line 158, revised manuscript)

*5) It is not reported the use of intravascular imaging (IVUS, OCT).*

Response

We have no data for imaging devices for PCI in the J-PCI registry, but referring to studies from Japan in the same period (Watanabe H, et al. JAMA 2019, Nakamura M, et al. Circ J 2020), it is estimated that imaging devices were used in more than 80% of PCI cases during this period. Moreover, the real-world use of IVUS was associated with reduction in coronary dissection, but not bleeding complications (Kuno T, et al. Heart an Vessels 2019). Therefore, the impact of the use of imaging devices on bleeding complications or subsequent fatal events may not be significant.

*6) It would be interesting to know if and which mechanical support (IABP, ECMO, Impella) was used in cardiogenic shock patients and how it could affect bleeding complications.*

Response

We also recognize that the data on the use of mechanical support against cardiogenic shock would help us further understand bleeding complications or subsequent fatal events. However, the J-PCI registry during this study periods does not have sufficient data on these mechanical devices for cardiogenic shock (the registry became mandatory in the middle of 2018).

Response to the Reviewer #2

*Reviewer #2*

*This paper reports results from the Japanese national PCI registry on bleeding complications from 388,866 procedures performed at 718 hospitals between 2017-2018. The authors found substantial variability in bleeding complications and mortality among the centers and provided some insights into associations of complications with center characteristics. The paper adds valuable information on complications from PCI in contemporary practice. The authors acknowledge the study limitations, i.e., observational study design, which limits conclusions on causal relationships. Foremost, the authors should be careful ascribing all issues to bleeding complications since the latter, of course, may just be a result from vascular injury not from excessive anticoagulation. The authors do address this to some extent by differentiating access site vs. other complications but nevertheless, further information is needed to understand the nature of complications leading to bleeding.*

Response

We appreciate the Reviewer’s helpful comments and agree that we should be careful about ascribing all issues to bleeding complications. We acknowledge that the causality between bleeding complications and in-hospital death cannot be proven. First of all, we had no data to what extent bleeding complications were fatal. The J-PCI registry’s definition of bleeding complications is largely equivalent to Bleeding Academic Research Consortium (BARC) 3A-C or above. Previous studies demonstrated that the ratio of fatal bleeding among patients with BARC 3A-C or above was approximately 10% (Vranckx P, et al. J Am Coll Cardiol 2016, Ratcovich H, et al. Am J Cardiol 2021, Gargiulo G, et al. J Am Coll Cardiol 2018). Unfortunately, direct information on fatal bleeding was not available on the registry, but we believe the rate of fatal bleeding would be roughly the same for our study. Second, failure to rescue, defined by all-cause death after major perioperative complications, is well established as an indicator of the surgical quality of care (Silber JH, et al. Med Care 1992). Moreover, institutional differences in failure-to-rescue rates underlie the wide variation in in-hospital mortality after surgery (Ghaferi AA, et al. N Engl J Med 2009). We think that the results in our study were equivalent to these reports, and this concept may be applied to PCI. Third, hemoglobin level and status of antithrombotic agent use (i.e., whether DAPT or Triple therapy) at the time of index PCI are crucial factors when considering bleeding complications. Based on these points, we have added the descriptions in the Results and Discussion sections as follows:

**Results**

**Table 1. Patient characteristics**

|  | **Overall** | **Very low mortality** | **Low mortality** | **Medium mortality** | **High mortality** | **Very high mortality** | ***P* value** |
| --- | --- | --- | --- | --- | --- | --- | --- |
|  |  |  |  |  |  |  |  |
| Cardiogenic shock within 24 hours | 13,419 (3.5) | 3,568 (3.3) | 2,710 (3.7) | 2,481 (3.6) | 2,513 (3.5) | 2,147 (3.1) | <0.001 |
| Baseline hemoglobin, g/dL | 13.2 ± 2.0 | 13.1 ± 2.0 | 13.2 ± 2.1 | 13.2 ± 2.0 | 13.2 ± 2.1 | 13.2 ± 2.1 | 0.002 |
|  |  |  |  |  |  |  |  |
| **Antithrombotic agents given at time of PCI** | | |  |  |  |  |  |
| Antiplatelet agents | 357,447 (92) | 97,149 (91) | 65,072 (89) | 63,565 (92) | 67,774 (95) | 63,887 (93) | <0.001 |
| Aspirin | 345,703 (89) | 93,953 (88) | 62,893 (86) | 61,652 (89) | 65,597 (92) | 61,608 (90) | <0.001 |
| Clopidogrel | 135,698 (35) | 37,721 (35) | 24,565 (34) | 26,661 (39) | 24,292 (34) | 22,459 (33) | <0.001 |
| Prasugrel | 191,263 (49) | 50,054 (47) | 35,993 (49) | 31,079 (45) | 37,380 (52) | 36,757 (54) | <0.001 |
| Ticagrelor | 468 (0.1) | 219 (0.2) | 94 (0.1) | 68 (0.1) | 53 (0.1) | 34 (0.05) | <0.001 |
| Anticoagulant agents | 26,309 (6.8) | 6,694 (6.3) | 4,950 (6.8) | 4,962 (7.2) | 5,129 (7.2) | 4,574 (6.7) | <0.001 |
| Warfarin | 10,378 (2.7) | 2,793 (2.6) | 1,972 (2.7) | 1,805 (2.6) | 2,014 (2.8) | 1,794 (2.6) | <0.001 |
| Dabigatran | 1,410 (0.4) | 348 (0.3) | 260 (0.4) | 297 (0.4) | 236 (0.3) | 269 (0.4) | 0.01 |
| Rivaroxaban | 5,004 (1.3) | 1,326 (1.2) | 834 (1.1) | 907 (1.3) | 1,026 (1.4) | 911 (1.3) | <0.001 |
| Apixaban | 5,388 (1.4) | 1,260 (1.2) | 1,078 (1.5) | 1,110 (1.6) | 1,037 (1.5) | 903 (1.3) | <0.001 |
| Edoxaban | 4,128 (1.1) | 1,046 (1.0) | 824 (1.1) | 799 (1.2) | 795 (1.1) | 664 (1.0) | 0.051 |
| Dual antiplatelet therapy | 316,061 (81) | 84,998 (80) | 58,456 (80) | 55,841 (81) | 59,763 (84) | 57,003 (83) | <0.001 |
| Triple therapy* | 19,205 (4.9) | 4,623 (4.3) | 3,727 (5.1) | 3,550 (5.1) | 3,951 (5.5) | 3,354 (4.9) | <0.001 |

Values are expressed as means ± standard deviation or numbers (%). Chronic kidney disease was defined as the presence of proteinuria, and/or a serum creatinine level ≥1.3 mg/dL, and/or an estimated glomerular filtration rate level ≤60 mL/min per 1.73 m^2^.

BMS, bare metal stent; CABG, coronary artery bypass grafting; COPD, chronic obstructive pulmonary disease; DCA, directional coronary atherectomy; DES, drug-eluting stent; LAD, left anterior descending artery; LCX, left circumflex artery; LMCA, left main coronary artery; NSTEMI, non-ST-segment elevation myocardial infarction; PCI, percutaneous coronary intervention; RCA, right coronary artery; STEMI, ST-segment elevation myocardial infarction; UA, unstable angina.

*Triple therapy indicates an anticoagulant agent plus dual antiplatelet therapy.

(Page 10, line 149–158, revised manuscript)

**Discussion**

“… Second, we cannot determine the causality between bleeding complications and death during hospitalization. However, failure to rescue, defined by all-cause death after major perioperative complications, is well established as an indicator of the surgical quality of care [19]. The results in our study were equivalent to this, and this concept may be applied to PCI. Third, blood transfusion practices and thresholds vary between hospitals [25], which may have affected our results. Fourth, the number of variables in the J-PCI registry is limited and measured or unmeasured confounders were present.”

(Page 16, line 260–266, revised manuscript)
